# Iodinin (1,6-Dihydroxyphenazine 5,10-Dioxide) from *Streptosporangium* sp. Induces Apoptosis Selectively in Myeloid Leukemia Cell Lines and Patient Cells 

**DOI:** 10.3390/md11020332

**Published:** 2013-01-30

**Authors:** Lene E. Myhren, Gyrid Nygaard, Gro Gausdal, Håvard Sletta, Knut Teigen, Kristin F. Degnes, Kolbjørn Zahlsen, Anders Brunsvik, Øystein Bruserud, Stein Ove Døskeland, Frode Selheim, Lars Herfindal

**Affiliations:** 1 Department of Biomedicine, University of Bergen, Jonas Lies vei 91, N-5009 Bergen, Norway; E-Mails: lene.myhren@biomed.uib.no (L.E.M.); gyrid.nygard@biomed.uib.no (G.N.); gro.gausdal@biomed.uib.no (G.G.); knut.teigen@biomed.uib.no (K.T.); stein.doskeland@biomed.uib.no (S.O.D.); frode.selheim@biomed.uib.no (F.S.); 2 SINTEF Materials and Chemistry, Department of Biotechnology, Sem Sælands vei 2a, N-7465 Trondheim, Norway; E-Mails: havard.sletta@sintef.no (H.S.); kristin.f.degnes@sintef.no (K.F.D.); kolbjorn.zahlsen@sintef.no (K.Z.); anders.brunsvik@sintef.no (A.B.); 3 Section for Haematology, Institute of Medicine, University of Bergen, Jonas Lies vei 91, N-5009 Bergen, Norway; E-Mail: oystein.bruserud@helse-bergen.no; 4 Department of Internal Medicine, Haukeland University Hospital, N-5021 Bergen, Norway; 5 Translational Signalling Group, Haukeland University Hospital, N-5009 Bergen, Norway

**Keywords:** acute myeloid leukemia, natural products, daunorubicin, patient samples

## Abstract

Despite recent improvement in therapy, acute myeloid leukemia (AML) is still associated with high lethality. In the presented study, we analyzed the bioactive compound iodinin (1,6-dihydroxyphenazine 5,10-dioxide) from a marine actinomycetes bacterium for the ability to induce cell death in a range of cell types. Iodinin showed selective toxicity to AML and acute promyelocytic (APL) leukemia cells, with EC50 values for cell death up to 40 times lower for leukemia cells when compared with normal cells. Iodinin also successfully induced cell death in patient-derived leukemia cells or cell lines with features associated with poor prognostic such as FLT3 internal tandem duplications or mutated/deficient p53. The cell death had typical apoptotic morphology, and activation of apoptotic signaling proteins like caspase-3. Molecular modeling suggested that iodinin could intercalate between bases in the DNA in a way similar to the anti-cancer drug daunorubicin (DNR), causing DNA-strand breaks. Iodinin induced apoptosis in several therapy-resistant AML-patient blasts, but to a low degree in peripheral blood leukocytes, and in contrast to DNR, not in rat cardiomyoblasts. The low activity towards normal cell types that are usually affected by anti-leukemia therapy suggests that iodinin and related compounds represent promising structures in the development of anti-cancer therapy.

## 1. Introduction

Acute myeloid leukemia (AML) is a hematopoietic stem cell disorder where the myeloid precursor cells have acquired mutations that impair apoptosis and differentiation and that confer proliferative and/or survival advantages. This causes excessive proliferation and rapid accumulation of myeloid precursor cells in the bone marrow. If left untreated, death occur within weeks or months after diagnosis. AML is a heterogeneous disease, with large variations in disease progression and therapy response. Two of the most common sub-types (WHO classification, 2008) are AML with recurrent cytogenetic abnormalities, and acute promyelocytic leukemia (APL), which have different treatment regimes. Whereas differentiation therapy, often in combination with cytostatics like arsenic trioxide, has proven successful for many APL cases [1], the treatment regime for AML often involves high doses of the cell cycle specific inhibitor cytarabine (Ara-C) in combination with the cell cycle unspecific inhibitor anthracycline daunorubicin (DNR) [2]. Complete remission is reached in 30%–40% of AML patients less than 60 years old, and less than 10% in patients older than 70 years [3]. However, relapse risk is in the range of 45%–50% in older patients, making AML the leading cause of death due to leukemia with a five-year relative survival below 20% [4,5]. Intensive chemotherapy is often severe and sometimes has lethal side-effects, such as lesions in hematopoietic tissue, particularly the bone marrow, as well as the intestine and the heart [6,7,8]. There is thus a need for novel compounds that selectively target leukemia blasts, and leave normal tissues and cells largely unaffected. 

Phenazines are nitrogen-containing heterocyclic compounds produced by a variety of bacteria. They represent a group of metabolites with potential for the discovery of new anti-infective agents, and so far, hundreds of the more than 6000 phenazine-containing compounds identified have biological activities, usually antibiotic properties [9]. However, their natural physiological function and mode of action still remains largely unknown [10]. The phenazine iodinin (1,6-dihydroxyphenazine 5,10-dioxide) was discovered to function as an anti-bacterial compound [11], and one study reported low activity against a mouse sarcoma model [12]. The aim of the present study was to elucidate the anti-cancer potential of iodinin, which was identified as a potent anti-cancer compound in a screen of marine actinomycetes bacteria. We found iodinin to be particularly potent against leukemia cell lines and AML-patient blasts, and it was less toxic than DNR towards peripheral blood leukocytes (PBL), rat cardiomyoblasts and blood platelets. These data suggest that iodinin or related compounds should be further investigated as potential lead structures for the development of drugs for AML treatment.

## 2. Results

### 2.1. Iodinin Shows High Selectivity towards Myeloid Leukemia Cells

**Figure 1:**
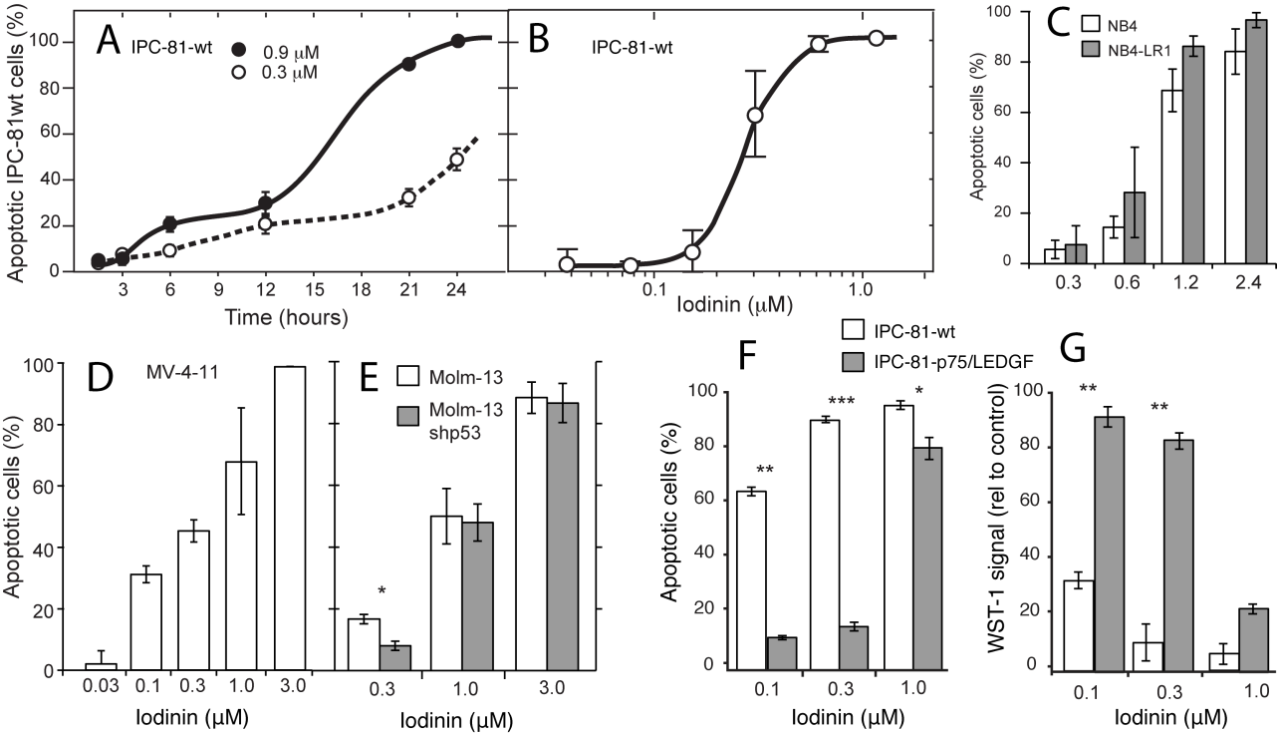
Iodinin is a potent cell death inducer in acute promyelocytic leukemia (APL) and acute myeloid leukemia (AML) cell lines. (**A**) Rat acute promyelocytic leukemia cells (IPC-81) were incubated with iodinin for various periods of time, fixed in buffered formaldehyde with the DNA-dye Hoechst 33342, and cell death was scored by differential interference contrast and UV-microscopy. (**B**–**E**) APL and AML cells were treated with increasing concentrations of iodinin for 24 h fixed in buffered formaldehyde and cell death was scored as described above. (**F** and **G**) IPC-81-wt cells or expressing p75/LEDGF were given daily pulses of anthracycline daunorubicin (DNR) for three days as described in the Experimental Section. The metabolic activity was measured by the WST-1 assay, and apoptosis as described for panel (**A**). Untreated cells or cells treated with solvent had always less than 4% apoptosis. The data are average and SEM of 3–5 experiments. Asterisks denote significance at *p* < 0.05 (*), <0.01 (**), <0.005 (***), *t*-test, for comparison of different strains of the same cell line (**C**, **E**, **F** and **G**).

**Figure S1:**
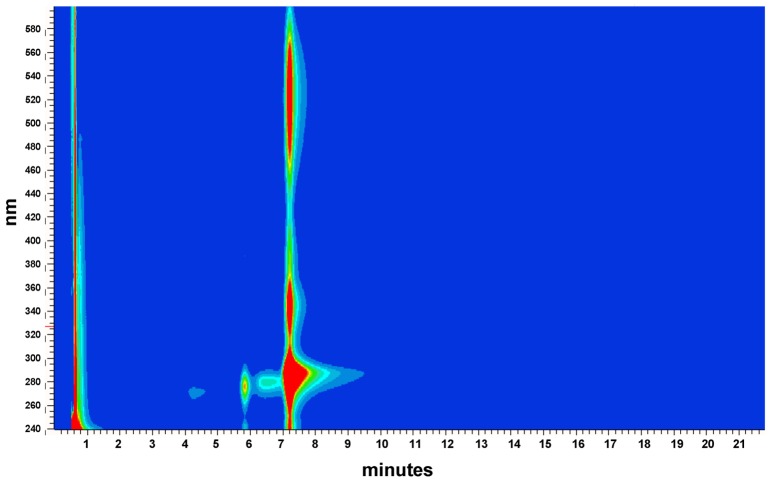
UV-DAD spectra of purified iodinin.

**Figure S2:**
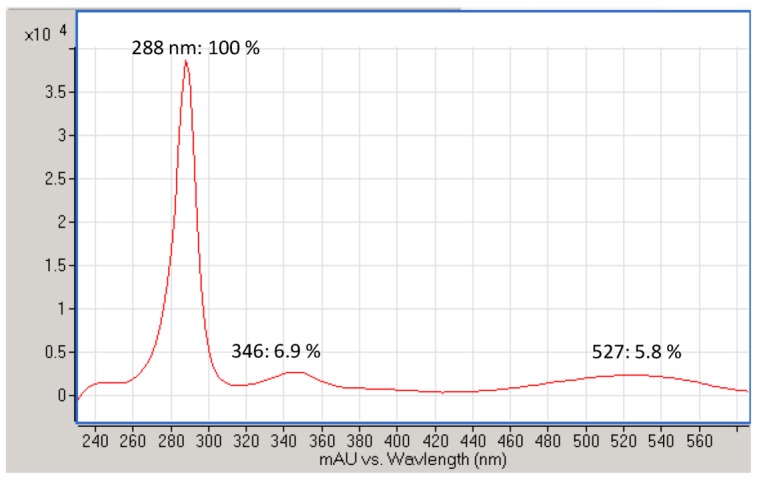
UV profile of purified iodinin produced by isolate MP55-27. UV absorption (nm) at UV max and % of UV max stated in the figure.

**Figure S3:**
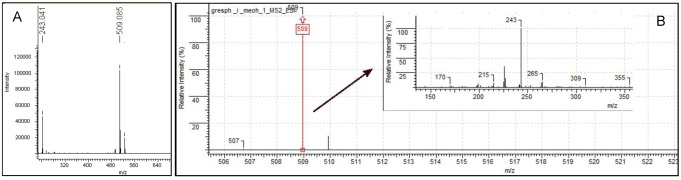
LC-TOF (**A**) and LC-Trap spectra (**B**) of iodinin. Two main ions were detected, and the accurate mass of the most abundant isotope peak in each spectrum is stated above each MS-spectrum. The two main ions represent iodinin monomer and the Na-adduct of the iodinin dimer. The spectrum inserted in (**B**) is the MS/MS spectrum of the Na-adduct of the iodinin dimer (*m/z* = 509).

Iodinin (1,6-dihydroxyphenazine 5,10-dioxide) produced from MP53-27 was identified by UV and MS-analyses (Supplementary Information Figure S1, Figure S2, Figure S3) and isolated to a chromatographic purity >90% (Figure S1) prior to cytotoxicity testing. We first tested iodinin for cytotoxicity against a panel of seven cell lines (Table 1), and found that iodinin showed selectivity towards the leukemia cells in the panel. Iodinin was, for instance, more than 15 times more effective towards the human lymphoid leukemia cell line Jurkat T than normal NRK fibroblasts (Table 1). We next tested iodinin against several AML cell lines (Table 1 and Figure 1).

**Table 1:** EC50 values (±SEM) of iodinin against various cell lines. The cells were treated with increasing doses of iodinin for 24 h before viability was assessed by microscopic evaluation of apoptosis and the WST-proliferation assay as described in the Experimental section. The data are based on regression analyses of 3 to 6 experiments.

Cell	Reference or ATTC No.	Origin	Disease	Features	EC50 (μM)
Primary heptaocytes		Rat		Freshly isolated primary hepatocytes in suspension	>5.0
Cardiomyoblasts	CRL-1446	Rat			>5.0
NRK	CRL-6509	Rat		Normal rat kidney fibroblasts	>10
Jurkat T, Clone E6-1	TIB-152	Human	Acute T-cell lymphoblastic leukemia		0.8 ± 0.2
SH-SY5Y	CRL-2266	Human	Neuroblastoma		2.7 ± 0.2
HeLa	CCL-2	Human	Cervical epithelial adenocarcinoma	Low levels of p53 expression	>10
U-87 MG	HTB-14	Human	Astrocytoma		>10
NB4	[13]	Human	Acute promyelocytic leukemia (APL)	t(15;17) (q22;q11-12) translocation, ATRA-induced differentiation	0.75 ± 0.13
NB4-LR1	[14]	Human	Acute promyelocytic leukemia (APL)	ATRA and cAMP needed to induce differentiation	0.70 ± 0.10
IPC-81	[15]	Rat	APL	Brown Norwegian rat myeloid leukemia model	0.24 ± 0.15
IPC-81 Bcl-2	[16]	Rat	APL	Enforced expression of Bcl-2	3.15 ± 0.15
Molm13	[17,18]	Human	Acute myeloid leukemia (AML)	ins(11;9)(q23;p22p23), FLT3 itd	1.0 ± 0.12
Molm13-SHp53		Human	AML	Silenced p53	1.0 ± 0.09
MV-4-11	[18,19]	Human	AML, myelomonocytic	t(4;11) translocation, FLT3 itd	0.50 ± 0.20

Among these were the IPC-81, NB4 and NB4-LR1 APL cells lines. Iodinin induced cell death at doses below 1 μM in all cell lines, being most potent against IPC-81 cells (EC50: 0.24 μM, Figure 1B). Bcl-2 appeared to protect cells against iodinin (Table 1), whereas p53 status did not seem to affect iodinin cytotoxicity (Figure 1E). Cell death did not occur until 6–12 h after addition of iodinin (Figure 1A). Chemotherapy with DNR is often given as short intravenous pulses for several consecutive days [2,20], and we wanted to know if the apoptosis-inducing effect of iodinin could be enhanced by a similar regime. By incubating IPC-81 cells with daily 2-h pulses with iodinin for three days, we were able to lower the EC50 value to 100 nM (Figure 1F,G). The same regime was tested on IPC-81 cells with enforced expression of the survival factor LEDGF/p75, a protein that is up-regulated in AML blasts from patients with chemo-resistant AML [21], and makes cells more resistant to DNR-induced cell death [21]. Iodinin (1 μM) also induced cell death in these cells (Figure 1F,G).

### 2.2. Iodinin Induces Cell Death with Apoptotic Features, and Shows Structural Similarity to Daunorubicin

**Figure 2:**
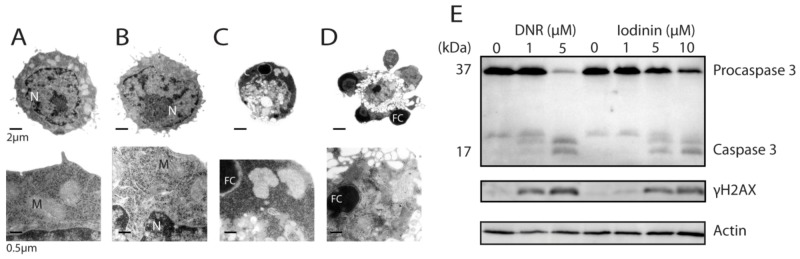
Iodinin induces apoptotic cell death in leukemia cells. (**A**–**D**) Transmission electron micrographs of IPC-81 cells treated with solvent (DMSO, **A**), or 0.3 μM of iodinin (**B**–**D**) for 21 h. N is nucleus, M is mitochondria, and FC is fragmented and condensed DNA. (**E**) Modulations of proteins in iodinin- and DNR-mediated leukemia cell death. NB4 cells were treated with the given concentrations of DNR or iodinin for 6 h. Cell extracts were immuno-blotted and probed for caspase 3, γH2AX, and β-actin, as described in the Experimental Section.

IPC-81 APL cells showed typical apoptotic features such as cell shrinkage, and chromatin condensation and fragmentation (Figure 2A–D). We noted also budding of apoptotic bodies containing organelles (Figure 2D), and that the mitochondria were apparently intact until the latest stage of cell death (Figure 2C,D). Further evidence for apoptotic cell death was present, such as cleavage of procaspase 3 to active pro-apoptotic caspase 3 [22] (Figure 2D). In line with this, we found that iodinin induced internucleosomal DNA-fragmentation in leukemia cells when apoptotic morphology was present (data not shown). Furthermore, both iodinin and DNR induced phosphorylation of histone H2AX in human NB4 cells (Figure 2D). H2AX phosphorylation and formation of γH2AX foci arises within a few minutes after DNA double stranded breaks an [23,24]. This is also an early event during anthracyclin-induced apoptosis [25].

**Figure 3:**
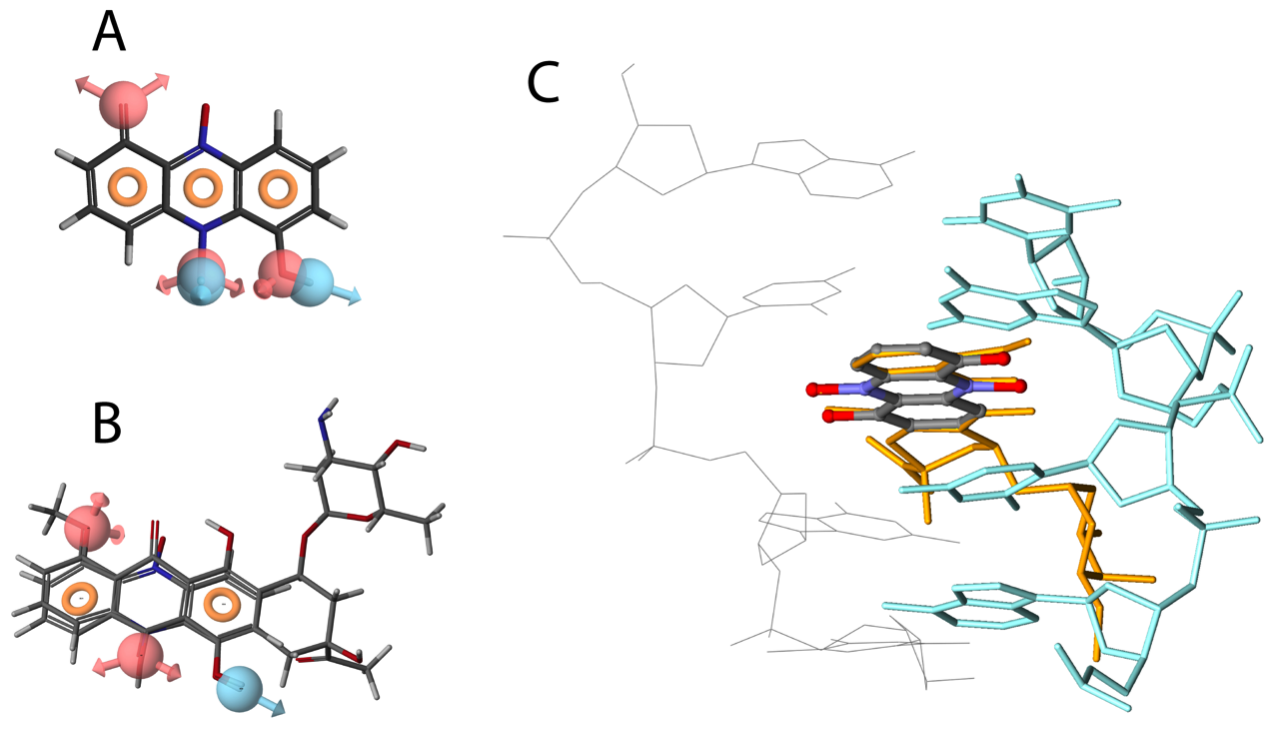
Structural similarities between iodinin and DNR. Pharmacophore model of iodinin (**A**), common pharmacophore features of iodinin and DNR (**B**), and suggested intercalation of iodinin (colored by atom type) with DNA (green and grey) (**C**). DNR is also shown in orange, overlapping with iodinin. The pharmacophore models were created with the Phase module of the Schrödinger™ software, whereas the suggested interaction of iodinin with DNA was prepared in Discovery Studio, based on the structural alignment (**B**) and a previously published interaction of DNR with DNA [27].

Previous studies have shown that many phenazines can interact with polynucleotides, and inhibit DNA template-controlled RNA synthesis [10,26]. We found a high degree of similarity between the pharmacophores generated for iodinin and DNR (Figure 3A,B), with two hydrogen-acceptors and one donor common in addition to the ring structures. Based on the alignment between DNR and iodinin (Figure 3B) we used the structure of DNR in complex with DNA [27] to prepare a model of how iodinin incorporates into DNA (Figure 3C) and cause double strand breaks as suggested by the phosphorylation of H2AX (Figure 2G). Although phenazines intercalate between the bases in the DNA, the pharmacophores generated for iodinin and DNR had higher similarity to each other than to those generated for a phenazine moiety (not shown). This suggests that the iodinin-DNR interaction is more similar to that with DNR than to phenazines. 

### 2.3. Iodinin Induces Cell Death in AML-Patient Blasts, but Has Low Toxicity to Cardiomyoblasts, Leukocytes and Platelets

**Figure 4:**
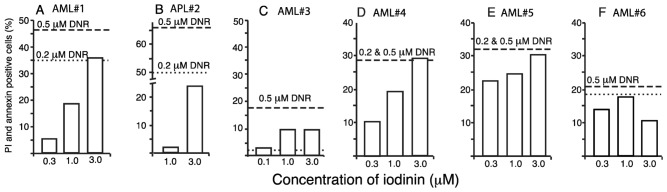
Iodinin induces apoptosis in leukemia patient blasts. Blasts isolated from peripheral blood samples from six leukemia patients were treated with iodinin (0.3, 1 or 3 μM) or DNR (0.2 or 0.5 μM) for 18 h. Samples were assessed for drug-induced cell death by FACS analysis of AnnexinV and PI labeling. The background (control) was subtracted from the data. See Table 2 for description of the patient samples.

To further explore the anti-leukemic potential of iodinin, we tested if iodinin could induce apoptosis in blasts from one APL and five AML patients (Table 2 and Figure 4). Except from blasts from patient AML#3 that appeared to be resistant to both DNR and iodinin, we found that both iodinin and DNR induced apoptosis in the patient blasts. In blasts from AML#1, AML#4 and AML#5, iodinin induced cell death in a dose responsive manner. Blasts from patient APL#2 responded well to DNR, but little to iodinin. The activity of iodinin towards these patient blasts suggests that iodinin could have potential as an anti-leukemic drug against some AML variants, even though it had lower efficiency than DNR. 

**Figure 5:**
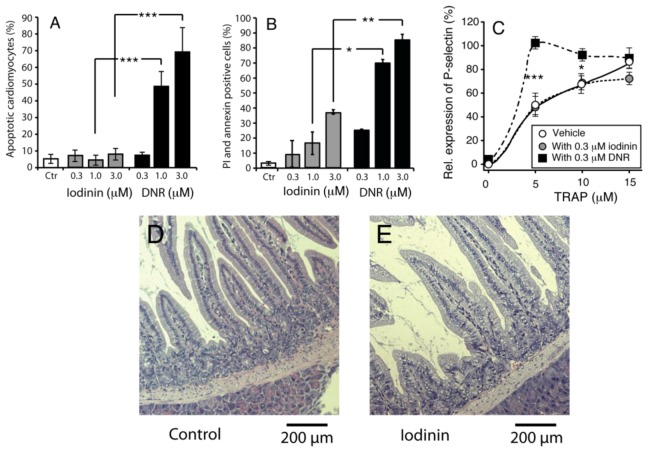
Iodinin exhibit low toxicity towards normal cells and tissues. (**A**) Rat cardiomyoblasts (H9C2) were incubated with iodinin or DNR for 24 h before viability was assessed by microscopic evaluation of surface morphology. (**B**) Peripheral blood leukocytes were incubated with the indicated concentrations of iodinin or DNR for 24 h before FACS analysis of AnnexinV and PI staining. (**C**) Blood platelets were incubated (15 min) with increasing concentrations of the thrombin receptor agonist peptide (TRAP) with or without iodinin or DNR and activation was determined as described in the Experimental section. Platelets incubated with 20 μM TRAP was set as 100% activation. The data are average of three to five experiments and SEM (**A**) and (**C**), or two experiments and each measurement (**B**). Asterisks denote significance at *p* < 0.05 (*), <0.01 (**), <0.005 (***), *t*-test in (**A**–**C**). (**D** and **E**) Histological appearance of duodenum of mice given vehicle, (**D**) or iodinin (10mg/kg, **E**) daily for three days. Organs were collected the day after the last treatment. See Experimental Section for further details.

Severe toxic side-effects are common in chemotherapy against leukemia. We found that iodinin was less toxic to both H9C2 rat cardiomyoblasts (Figure 5A) and peripheral blood leukocytes (PBL, Figure 5B). In addition, iodinin did not modulate blood platelet activation, measured as externalization of P-selectin (Figure 5C). Chemotherapy can cause severe side effects on several tissues; the earliest signs of damage are bone marrow depletion and intestinal mucositis [28,29]. We treated mice with iodinin, and looked for signs of damage on tissues normally affected by chemotherapeutics. We found no signs of iodinin-induced damage in the tissues analyzed (heart, spleen, stomach, duodenum, kidneys, liver, bone marrow) (Figure 5D,E and not shown). Intestinal mucositis and bone marrow depletion is usually seen during or just after chemotherapy [28,29]. Moreover, we found no signs of toxicity in mice up to three weeks after administration of iodinin (not shown).

**Table 2:** AML patient characteristics. APL and AML blasts were isolated from 6 patients. Patient age and sex are listed, together with FAB classification, cytogenetic findings, and Flt3 and NPM1 mutation state of their isolated blasts. ITD = internal tandem duplication, ins = insertion, nd = not determined. Patient AML#1 and AML#6 reached complete haematological remission after two induction cycles (cytarabine in combination with an anthracycline), whereas patients AML#3 and AML#5 reached complete remission after the first induction cycle. AML#4 relapsed 11 months after first remission and was given 22 rounds of intensive chemotherapy (cytarabine), but second remission was not reached. Blasts from patient AML#4 were obtained after diagnosis of relapse. APL#2 was treated according to the regimen described by Fenaux *et al.* [1], and reached haematological remission after the induction cycle, and was in molecular remission after the second consolidation cycle before start of maintenance therapy.

Patients	Age	Sex	Cytogenetics	FABclassification	FLt3	NPM1
AML#1	24	M	Multiple	M2	wt	wt
APL#2	39	M	T(15;17)	M3	wt	wt
AML#3	48	M	Inv(16)	M4	wt	wt
AML#4	29	M	Normal	M4	ITD	Ins
AML#5	18	F	Inv(16)	M4	wt	wt
AML#6	29	F	Normal	M5	wt	nd

## 3. Discussion

The present study demonstrates that iodinin is particularly potent against myeloid leukemia cells compared with other cancer and normal cells (Table 1, Figure 1, Figure 4 and Figure 5). It was noteworthy that iodinin induced apoptosis also in cells with mutations linked with poor outcome prognosis, such as enforced expression of p75, Flt3 internal tandem duplication (ITD) and ATRA-resistance (Figure 1). Moreover, blasts from patient AML#4 showed a dose-dependent response to iodinin, but not to DNR (Figure 4D). These blasts have Flt3 ITD mutation, associated with poor outcome, and the patient suffered from relapse that was not diminished after intensive chemotherapy (Table 2). The blasts from AML#1 showed a dose-response to both iodinin and DNR. This patient had multiple chromosomal abnormalities, which is considered unfavorable (for reviews on cytogenetics in AML and risk, see [30,31]). Although more cell lines and patient blasts are needed to obtain an overview of which sub-types of AML iodinin can be effective against, we conclude that iodinin shows anti-leukemic activity based on its ability to induce apoptosis in cells with several mutations that are associated with poor disease outcome. Iodinin induces an apoptotic phenotype, with activation of pro-apoptotic signals (Figure 2), that could be inhibited by enforced expression of the anti-apoptotic protein Bcl-2 (Table 1), suggesting that iodinin triggers fundamental death pathways in the leukemic cells. We noticed that cells treated with iodinin for a short period of time followed by washing still underwent apoptosis (Figure 1F,G). This points towards not only rapid uptake of iodinin by the leukemia cells, but also that it is retained inside the cells for instance by binding to DNA (Figure 3) or other cellular components.

In addition to being a potent apoptosis inducer in many leukemia cell lines (Figure 1), iodinin proved to have low toxicity towards non-AML cells like primary rat hepatocytes and NRK-cells (Table 1) as well as rat cardiomyoblasts, PBL and blood platelets (Figure 5). The three latter are all susceptible to DNR-induced toxicity [6,7,8,28,32]. Interestingly, iodinin showed no adverse effects in cardiomyoblasts, whereas DNR induced 50% cardiomyoblast death at 1 μM (Figure 5A), and DNR was about five times more toxic than iodinin against PBL (Figure 5B). This indicates that normal cells and tissues have higher tolerance to iodinin than DNR. The low toxicity of iodinin was also demonstrated in mice. There were no signs of damage in tissues excised from mice treated with 10 mg/kg iodinin for three days (Figure 5E), and mice appeared healthy three weeks after iodinin administration. These preliminary results suggest that iodinin exhibit low toxicity also *in vivo*.

Natural compounds have for long been recognized as a prolific source for potential anti-cancer drugs, and the number of drug candidates from natural origin increases [33]. Their potency is often caused by activation of the cell death machinery [34,35], interference with mitotic machinery [36], or by indirect initiation of cell death by, e.g., DNA-breaks or mitochondrial damage [37]. We present evidence suggesting that iodinin initiates apoptotic cell death by causing DNA-breaks (Figure 3B), which is similar to many anti-cancer drugs currently used today [38]. We therefore believe that it can be worth to pursue iodinin as an anti-leukemic lead compound. 

The next obstacle to overcome is the poor solubility of iodinin in aqueous media, caused by a lattice formed by strong hydrogen bonds between the molecules [39]. The hydrogen donors and acceptors responsible for this appear to partly define the drug characters of iodinin (Figure 3), and chemical modification of iodinin at these sites could attenuate its anti-leukemic activity. Although the solubility of iodinin in aqueous media is low, it is sufficient for intravenous infusion therapy similar to what is done with other anti-leukemic drugs [20]. Moreover, the recent advances in nanonization [40] paves way for the use of drugs with high melting temperature and low water solubility. If the apparent *in vivo* drug availability can be improved, we believe that iodinin and related compounds can prove to be valuable leads for treatment of AML, particularly in patients who tolerate conventional therapy poorly. 

## 4. Experimental Section

### 4.1. Purification and Identification of Iodinin from Isolate MP53-27

The actinomycete isolate MP53-27 was mass cultured in 1000 mL batches with medium consisting of oatmeal (30 g/L), malt extract (5 g/L), yeast extract (3 g/L), MgSO_4_·7H_2_O (0.4 g/L), NaCl (1 g/L), CaCO_3_ (5 g/L), glycerol (30 g/L), soluble starch (30 g/L) and glucose (30 g/L), with pH of 7.2. The biomass in the production culture was harvested by centrifugation and the pellet was freeze-dried. The freeze-dried pellet was homogenized with magnetic iron beads and extracted with 400 mL DMSO/g together with glass beads (1 mm) for 1 h. The cell pellet was removed by centrifugation followed by filtration to remove all insoluble matter. The clear supernatant was added an equal amount of water and kept on the bench for 30 min in order to precipitate iodinin. The precipitate was collected by centrifugation, washed with water to remove remaining DMSO and freeze-dried. The crude product was dissolved in DMSO and purified by reverse-phase HPLC, using an Agilent 1100 series preparative HPLC with fraction collection system with a 21 × 250 mm Zorbax SB-CN-column. 10 mM ammonium acetate pH 4 and methanol was used as mobile phases. The methanol was removed from the LC-fractions using a SpeedVac at 50 °C, and the precipitate washed with water. The isolated iodinin was freeze-dried and stored at −80 °C.

LC-DAD-TOF analyses of purified iodinin (Figure S1) were done on an Agilent LC system with a Zorbax Bonus-RP column (2.1 by 50 mm, 3.5 μm) connected to a G1315B DAD and a G1969 time-of-flight (TOF) apparatus to determine the accurate mass and UV-profile of the bioactive compound. The mobile phase was 10 mM ammonium acetate (pH 7) and acetonitrile. Electrospray ionization was performed as described previously [41]. Trap MS was performed on an Agilent G2445D IonTrap instrument equipped with electrospray ion source. IonTrap MS and MSMS experiments were performed by infusion of DMSO extracts diluted in methanol. 

### 4.2. Cell Maintenance and Experimental Conditions

The cell lines are described in Table 1. The NB4, Molm-13 and Jurkat T leukemia cell lines were cultured in RPMI medium enriched with 10% foetal bovine serum (FBS, Invitrogen, Carlsbad, CA, USA). IPC-81 cells were cultured in Dulbecco’s Modified Eagles Medium (DMEM) enriched with 10% horse serum (Invitrogen, Carlsbad, CA, USA) and MV4-11 were cultured in Iscove’s medium added 8 mM L-glutamine and 10% FBS. HeLa human cervical epithelial adenocarcinoma cells, U-87 MG human glioma, NRK normal rat kidney epithelial cells and H9C2 rat cardiomyoblasts were cultured in DMEM medium enriched with 10% FBS. All cell lines were cultured in media supplemented with 100 IU/mL penicillin and 100 mg/mL streptomycin (both from Cambrex, Verviers, Belgium) in a humidified atmosphere (37 °C, 5% CO_2_). All culture media were from Sigma (Sigma, La Jolla, CA, USA). 

For cytotoxic testing, the cells were seeded in 96 well tissue culture plates at 150,000 cells/mL (NB4, NB4-LR1, Jurkat-T, Molm13, MV4-11, IPC-81 wt and IPC-81 Bcl-2) or 50,000 cells/mL (SH-SY5Y, U-87 MG, NRK, H9C2). The adherent cell lines were left over night to attach to the substratum before experiments. The cells were exposed to various concentrations of iodinin for 24 h before assessment of viability by the reporter dye WST-1 (except for the H9C2 cardiomyoblasts) as described by the supplier (Roche Diagnostics, Basel, Switzerland). The cells were next fixed in 2% buffered formaldehyde (pH 7.4) with the DNA-specific dye Hoechst 33342 (Polysciences Inc., Eppelheim, Germany) and scored for apoptosis and necrosis as previously described [42,43]. EC50 values were determined by analyses of WST-1 data and microscopic evaluation and these data gave consistent dose-response curves (see Figure 1F,G). To mimic DNR-therapy, cells were exposed to iodinin for 2 h, washed and incubated in fresh medium for 22 h before another 2-h iodinin treatment followed by wash and a final 22 h incubation. Apoptosis was then assessed as described. Extraction of DNA and agarose electrophoresis of internucleosomal DNA fragmentation was as described in [44]. Tests for significance (ANOVA or student-*t*) were performed in SPSS statistical software [45]. 

### 4.3. Transmission Electron Microscopy

IPC-81 cells were treated with vehicle or 0.3 μM iodinin for 18 h before fixation in 1.5% glutaraldehyde in buffer (0.1 M sodium cacodylate, 0.1 M sucrose, 2.5 mM CaCl_2_, pH 7.4) for 20 min. They were further processed for TEM as described [46]. The specimens were examined using a Jeol JEM-1230 transmission electron microscope (JEOL Ltd., Tokyo, Japan). 

### 4.4. Western Blotting

NB4 cells treated with iodinin, daunorubicine or vehicle for 6 h were lysed in lysisbuffer (10 mM K_2_HPO_4_, 10 mM KH_2_PO_4_, 1 mM EDTA (pH 6.8) containing 10 mM CHAPS, 50 mM NaF, 0.3 mM NaVO_3_ and Complete mini protease inhibitor (Roche Diagnostics, Mannheim, Germany)), homogenised and centrifuged (13,000 rpm, 30 min, 4 °C). Protein lysates (50 μg) were separated by SDS-PAGE (5% stacking gel and 7.5 or 12.5% resolving gels) and blotted onto a polyvinyldifluoride membrane (Hybond, Amersham Biosciences, Freiburg, Germany). Primary antibodies were from Santa Cruz Biotechology (Santa Cruz, CA, USA; γH2AXgamma, caspase 3), Abcam (Cambridge, UK; actin), and secondary alkaline-phosphatase-conjugated antibodies (a-3687 and a-3562) were from Sigma. CDP-Star substrate was from Tropix (Bedford, MA, USA). Chemiluminescence was detected using a Luminescent Image Analyser Aparatus (LAS 3000, FujiFilm, Tokyo, Japan) and Image Gauge Software (FujiFilm, Tokyo, Japan).

### 4.5. Molecular Visualisation

Pharmacophore models were prepared with the Phase [47] module of the Maestro software [48]. Low energy conformations of DNR and iodinin tautomers at neutral pH were generated and pharmacophore sites were defined with default settings. The structures were then aligned based on the pharmacophores with the highest survival score. Structural alignment of iodinin and DNR in DNA was based on the generated pharmacophore model as well as previous studies [27] and visualized with the Discovery Studio 3.1 software [49].

### 4.6. Isolation of Blood Platelets and Measurement of P-Selectin Translocation

Freshly drawn venous blood was provided by the Blood Bank (Haukeland University Hospital, Bergen, Norway), and isolated as previously described [50,51]. In brief, blood was collected into a final 0.15 volume of acid citrated dextrose (ACD; 71 mM citric acid, 85 mM Na3-citrate, 100 mM glucose). Platelet-rich plasma (PRP) was obtained by centrifugation and transferred into a Ca^2+^-free Tyrode’s buffer by gel filtration through a Sepharose CL-2B gel matrix (Pharmacia Biotec, Uppsala, Sweden). The concentration of gel filtrated platelets (GFP) was measured by a ZM Coulter Counter (Coulter Electronics Ltd., Luton, UK) and adjusted to 3.5 × 10^8^ platelets/mL with Tyrode’s buffer. 

Flow cytometric measurement of P-selectin translocation to the platelet surface was performed as previously described [52,53]. GFP in PBS were incubated with vehicle, DNR or iodinin, and R-phycoerythrin (R-PE)-conjugated anti-human CD62 (BDIS, San Jose, CA, USA). After 15 min of pre-incubation, the samples were stimulated for 20 min with various concentrations of the synthetic thrombin receptor agonist peptide (TRAP, SFLLRN) from the Biotechnology center of Oslo (Rikshospitalet, Oslo, Norway). The level of P-selectin translocation was assessed by flow cytometric analysis using a FACSort Flow Cytomter and CellQuest Software from BDIS as previously described [53].

### 4.7. Flow Cytometry of AML Patient Material

The collection of patient cells was approved by the regional research ethics committee (Health Region III, Bergen, Norway) and conducted in accordance with the Declaration of Helsinki. Samples were collected after informed consent, and stored in biobanks approved by the Norwegian Royal Ministry of Health and the Norwegian Directorate for Health and Social Affairs. Primary peripheral blood mononuclear cells were isolated by density gradient separation (Ficoll-Hypaque; NyCoMed, Oslo, Norway); the percentage of leukemic cells after gradient separation exceeded 95% for all patients. For patient details, see Table 2. Normal peripheral blood leukocytes (PBL) were isolated from blood from healthy donors (Blood bank, Haukeland University Hospital, Bergen, Norway) as described in [54].

Patient cells were stored frozen in liquid nitrogen [55]. After thawing, blasts were suspended at 0.5 × 10^6^ cells/mL in Stem Span medium (Stem Cell Technologies, Vancouver, Canada) or RPMI with 10% FBS (peripheral blood leukocytes) before addition of drugs. Drug induced cell death was assessed by flow cytometric analysis of cells stained with AlexaFluor 647-AnnexinV (Molecular Probes) and propidium iodide (PI). At least 30,000 non-gated events were collected for each sample on an AccuriC6 (Ann Arbor, MI, USA). Non-specific cell death was subtracted. Drug-induced cytotoxicity increases the number of cells positive to both AnnexinV and PI.

### 4.8. Histopathological Analysis

NOD/SCID/B2mnull mice were given daily doses of vehicle or iodinin orally, or DNR i.v. through the tail vein for three days. Tissues and organs were excised from anesthetized mice, washed in ice-cold PBS and fixed in 2% buffered formalin (pH 7.4). The samples were embedded in paraffin, sectioned (2 μm) and stained with haematoxylin- and eosin. Some mice were given iodinin and kept for three weeks before being euthanized and checked for signs of tissue damage. Both groups of mice were weighed before and after administration of iodinin, but no weight loss was observed during the experiment.

Animals were kept in arrester particulate filtered cages during the experiments, and were given water and food *ad libitum*. The experiments were approved by the Norwegian Animal Research Authority and conducted according to the European Convention for the Protection of Vertebrates Used for Scientific Purposes.

## 5. Conclusions

In this article, we wanted to explore the marine natural compound iodinin as a possible anti-leukemic drug. Based on the high selectivity towards leukemia cells from the myeloid lineage (Table 1, Figure 1, Figure 4) compared to non-AML cells (Table 1, Figure 5), we conclude that iodinin has potential as a anti-leukemic lead compound. Although DNR had higher potency than iodinin against AML patient blasts, iodinin was significantly less toxic against relevant normal cell lines (Table 1, Figure 5) with an apparent therapeutic window that is comparable to many anti-cancer drugs in use. There are several examples of leads originated from natural sources that have been modified hemisynthetically to yield compounds with higher bioactivity or specificity, which could also be applied in cancer therapy [56,57]. We therefore believe that iodinin and related natural compounds could also prove to be important lead structures in the development of novel drugs. 

## Supplementary Information

Iodinin (1,6-dihydroxyphenazine 5,10-dioxide) was isolated and purified to >90% chromatographic purity (Figure S1) from freeze-dried cell pellets (also containing precipitated product) from MP53-27 cultures. Extracts and purified material from MP53-27 were analysed by two different MS techniques, LC-DAD-TOF and LC-IonTrap. The LC-DAD-TOF results showed a significant UV absorption peak at 288 nm, and smaller peaks at 346 and 527 nm (Figure S2) with dominating masses in ESI negative mode at *m/z* 243.040 *m/z* 509.073 (Figure S3A). The Trap MS experiments were performed in parallel (Figure S3B). In these experiments, fragmentation patterns confirmed that both the ion *m/z* 243.040 and *m/z* 509.073 were associated with the chromatographic peak corresponding to the bioactive compound (Figure S3B).

These results could only be explained if the molecular formula of the bioactive compound was C_12_H_8_N_2_O_4_, the accurate mass of the protonated molecule was M = 244.0484, and the ion *m/z* 509.073 was identified as a complex of two iodinin molecules associated with one Na^+^. Complexes, adducts and dimers are frequently seen in electrospray mass spectrometry [58] and it has been shown that polar *N*-oxide groups (present in iodinin) may form intermolecular hydrogen bonds with the hydroxyl groups [59].

Database search with the monomer accurate mass and UV spectrum [60] supported our conclusion that the bioactive compound identified in our study was iodinin.
